# Red beet betalains extraction process: A comprehensive review of methods, applications, and physicochemical properties

**DOI:** 10.1002/fsn3.4458

**Published:** 2024-09-20

**Authors:** Saba Eyshi, Nazila Ghareaghajlou, Mohammad Reza Afshar Mogaddam, Zahra Ghasempour

**Affiliations:** ^1^ Students Research Committee, Department of Food Science and Technology, Faculty of Nutrition and Food Sciences Tabriz University of Medical Sciences Tabriz Iran; ^2^ Food and Drug Safety Research Center Tabriz University of Medical Sciences Tabriz Iran; ^3^ Nutrition Research Center, Department of Food Science and Technology, Faculty of Nutrition and Food Sciences Tabriz University of Medical Sciences Tabriz Iran

**Keywords:** beetroot, betalain, betanin, degradation, efficiency, novel extraction

## Abstract

Red beet extract is rich in bioactive compounds and possesses health‐promoting properties. Moreover, the stability of red beet extract over a broad acidic pH range has given them great potential in developing new functional foods and drinks. The choice of extraction solvent and methodology significantly influences the efficiency of betalain extraction from plant vacuoles. Although the conventional solvent extraction method has been widely employed for betalain extraction, recent innovations have introduced alternative methods that offer advantages, such as reduced solvent consumption, energy efficiency, and minimized exposure to high temperatures. This paper aims to summarize the current knowledge about conventional and novel extraction methods, applications, biological activities, and purification of red beet betalains. Furthermore, the physicochemical properties of betalain‐rich extract of red beet and associated safety considerations have been investigated.

## Graphical abstract

Hydro‐alcoholic solutions extracted more betalains from red beet compared to water. Moreover, novel extraction methods were more efficient than conventional ones for the extraction of red beet betalains. Red beet betalains have gained attention due to their health‐promoting advantages and applications.

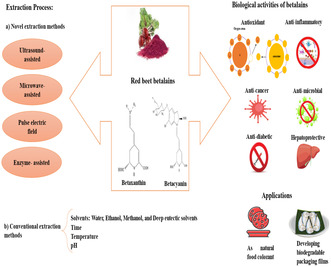

## 1 INTRODUCTION

Color, as one of the significant quality properties of foods, affects consumer acceptance of food products (Cömert et al., 2020). Safety problems of synthetic colorants, increased knowledge of consumers, and the availability of natural colorants have increased demand for the application of extracts from plants and vegetables (Farooq et al., 2020). The main plant‐derived pigments are betalains, anthocyanins, carotenoids, and chlorophylls (Ghareaghajlou et al., 2021). Since the color of betalains remains stable over a wide pH range, they are more efficient than anthocyanins for the coloration of low‐acidic foods (Agrawal, 2013).

**FIGURE 1:**
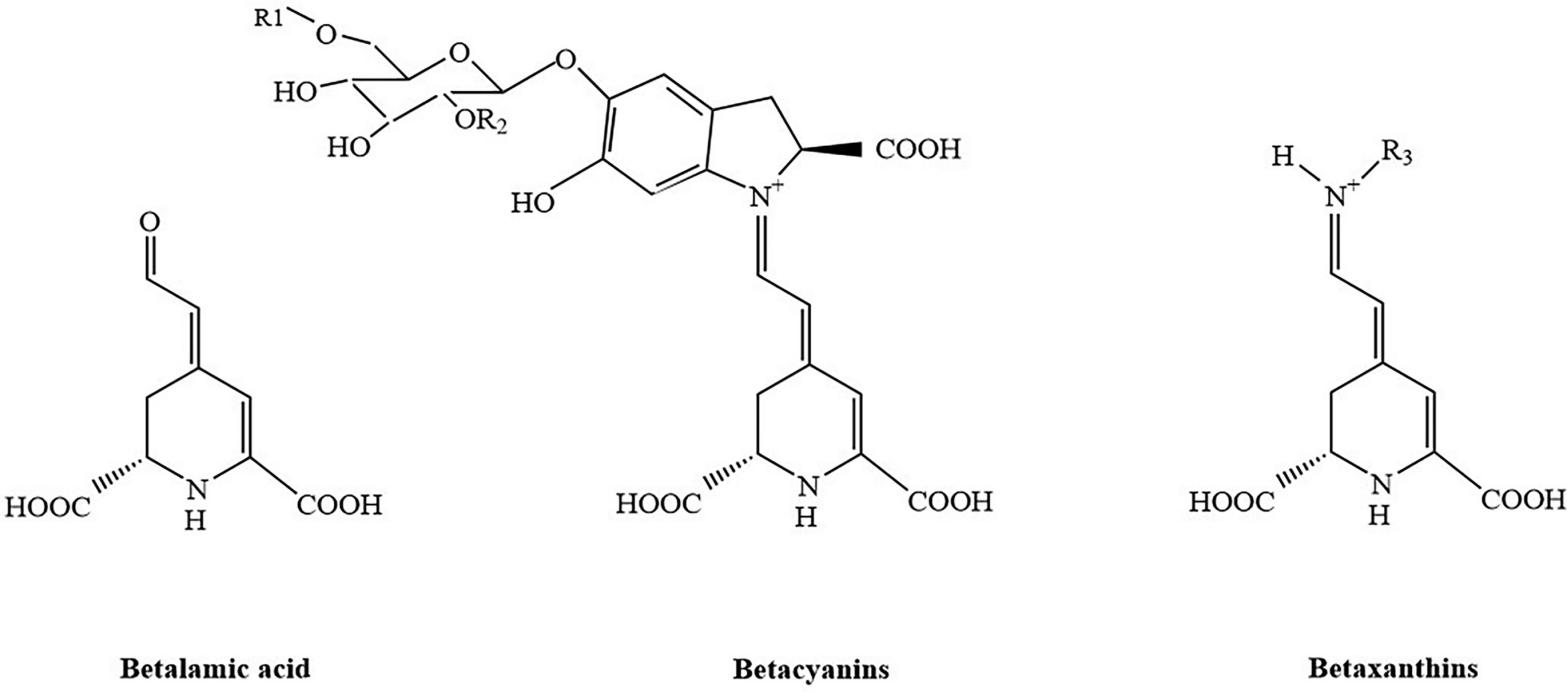
Schematic structures of betalamic acid, betacyanins, and betaxanthins.

Betalains are nitrogenous water‐soluble pigments derived from the amino acid tyrosine (Moreno‐Ley et al., 2021). Betalamic acid is the chromophore that exists in all betalains (Carrillo‐López & Yahia, 2017). The structural division of betalains into betacyanins and betaxanthins is determined by the residue added to betalamic acid (Azeredo, 2009; Gengatharan et al., 2015). Betacyanins and betaxanthins are formed when betalamic acid is attached to the molecules of *cyclo*‐3,4‐dihydroxyphenylalanine (*cyclo*‐DOPA) and amino acids (or amines), respectively (Sawicki, Bączek, et al., 2016). Betaxanthins are yellow‐colored pigments, including indicaxanthin and vulgaxanthin I and II, whereas betacyanins are red‐violet pigments, including betanin, prebetanin, isobetanin, and neobetanin (Fu et al., 2020). The chemical structures of betalamic acid, betacyanins, and betaxanthins are depicted in Figure 1. Betalains are found in edible portions of plants, leaves, and stems (Gandía‐Herrero et al., 2016). Red beet is the richest source of betalain pigments (Sawicki, Bączek, et al., 2016).

Red beet is cultivated in several countries worldwide (Kale et al., 2018). Red beet betalains in plant vacuoles exhibit health‐promoting advantages, including antioxidant, anti‐inflammatory, anti‐cancer, anti‐diabetic, antimicrobial, and hepatoprotective activities (Fernando et al., 2021; Hadipour et al., 2020; Sokolova et al., 2022). The amount of these pigments is associated with maturation, variety, and climatic conditions (Janiszewska, 2014). The color intensity of red beet betalains is mainly related to the ratio of betacyanins to betaxanthins (Lazăr et al., 2021). While red beet betacyanins are mainly betanin and isobetanin, the major betaxanthins are vulgaxanthin I and II. About 95% of red beet betalains are betanin, isobetanin, and vulgaxanthin I (Cardoso‐Ugarte et al., 2014).

The extraction process is accelerated using a combination of solvents, such as methanol, ethanol, and acetone. (Ghareaghajlou et al., 2021). During the extraction process, the solvent spreads into the plant tissue, solubilizes the betalain pigments, and spreads them out (Bastos & Gonçalves, 2017b). Selecting a suitable solvent for extraction requires considering its polarity, safety, cost, and availability (Fernando et al., 2021). Although the conventional solvent extraction method is commonly applied to extract betalains (Sagita, 2021), novel extraction methods can recover these pigments more efficiently (Zin, Anucha, et al., 2020b). Ultrasound‐assisted extraction (UAE) (Tabio‐García et al., 2021), supercritical fluid extraction (SFE; Fathordoobady et al., 2016), pulsed electric field (PEF) processing (Nowacka et al., 2019), microwave‐assisted extraction (MAE; Cardoso‐Ugarte et al., 2014), high hydrostatic pressure extraction (HHPE; Jiménez‐Aguilar et al., 2015), enzyme‐assisted extraction (EAE; Lombardelli et al., 2021), and pressurized liquid extraction (PLE; Gómez‐López et al., 2022) are novel extraction methods for extracting betalains. Additionally, ionic liquids (ILs), deep eutectic solvents (DES), and natural deep eutectic solvents (NADES) can be used to overcome some of the disadvantages of conventional solvents (Gomez et al., 2018; Huang et al., 2020; Santos et al., 2022).

There has not been a comprehensive investigation into various extraction and recovery methods of red beet betalains. Therefore, this research aims to investigate the influence of extraction methods on the quantity of red beet betalains and any associated safety considerations. Additionally, this study provides an overview of recovery methods, physicochemical properties, biological activities, and potential applications of red beet betalains.

## 2 THE EXTRACTION METHODS AND APPLICATIONS OF RED BEET BETALAINS

### 2.1 Conventional solvent extraction

**FIGURE 2:**
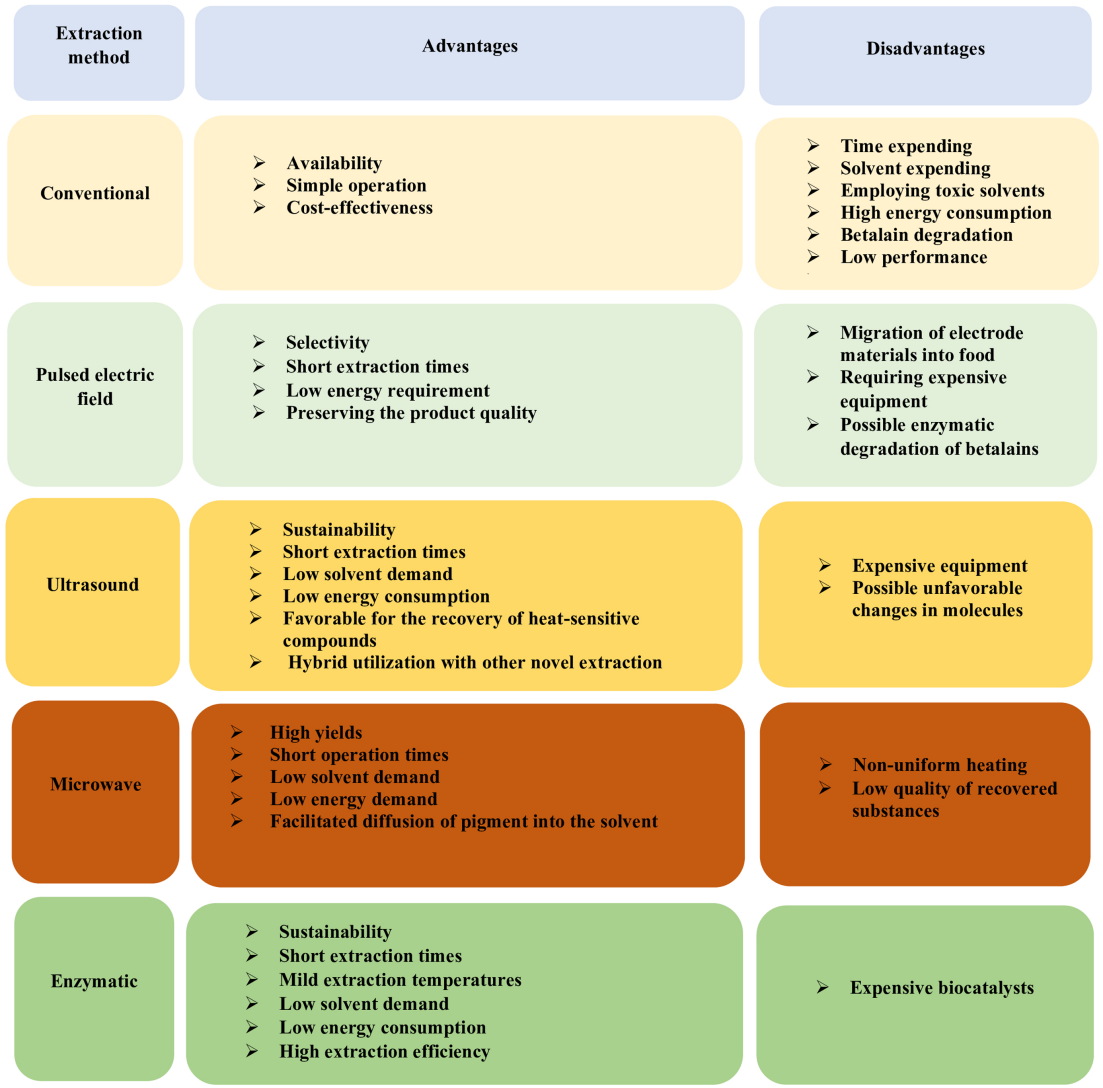
Advantages and disadvantages of conventional and novel extraction methods.

This method applies heating or agitation to dissolve solid materials with an appropriate volume of solvent (Zin et al., 2021). The conventional solvent extraction method is an accessible (Rosa et al., 2023), simple process (Kaderides et al., 2024), with low economic costs (Lazăr et al., 2021). However, this method requires high volumes of solvents, which are characterized by their high volatility, flammability, and toxicity (Santos et al., 2022). Moreover, the prolonged extraction time of this method leads to high energy consumption (Tabio‐García et al., 2021), betalain degradation (Hazervazifeh et al., 2023), and consequently, the low extraction efficiency of these pigments (Visockis et al., 2021; Figure 2).

The extractability of betalain pigments from red beets can be significantly enhanced by choosing the right solvent and adjusting extraction conditions, such as pH and temperature (Zin et al., 2021). Table 1 presents the various solvents used to extract red beet betalains.

**TABLE 1:** Different solvents used for the extraction of red beet betalains.

Solvent	Solid: liquid ratio	Extraction conditions	Results		References
			Optimized betalain concentration	Optimum conditions	
30% ethanol 50% ethanol 70% ethanol Distilled water	1:100	Agitation for 10 s Centrifugation of homogenate at 6000 rpm for 10 min Collection of supernatant Repetition the same procedure 2 more times	—	30% ethanol had the most betalain content	Ravichandran et al. (2013)
Citrate–phosphate buffer (pH 6.5) Water	1:200	Washing, peeling, slicing, and freeze‐drying of beetroots Using the dried powder for extraction	5.41 mg/g of betacyanins, 3.21 mg/g of betaxanthins, and 8.60 mg/g of total betalains were recovered by water. 4.98 mg/g of betacyanins, 3.12 mg/g of betaxanthins, and 8.10 mg/g of total betalains were recovered by citrate–phosphate buffer	Water extracted the highest amount of betacyanins, betaxanthins, and total betalains	Castellanos‐Santiago and Yahia (2008)
A mixture of ethanol: water (1:1)	0.1:25	Continuous shaking at a constant speed at a temperature of 25°C Further conventional extraction at a high temperature of 80°C	The extraction percentages of 20% and 10% were obtained at 80°C and 25°C, respectively	Betalain recovery at 80°C was more than room temperature	Cardoso‐Ugarte et al. (2014)
Citric acid solution (2%) Water	1:3	Washing and cutting red beets into cubes Blending with solvent for 1 min Filtration using filter paper	6.09 and 17.51 mg/100 mL of betanins were obtained by citric acid solution and water, respectively	The highest amount of betanins was obtained at the temperature of 50°C, time of 5–15 min, pH of 6 and 2 for water, and citric acid extract, respectively	Halwani et al. (2018)
9%–60% ethanolic solutions acidified with citric acid solutions (0.03%–2%)	1:10	Extraction at temperatures of 3–87°C for 3–74 min on a shaker at 100 rpm Centrifugation of the samples at 4°C for 10 min at 14,000 rpm. Separation of the supernatant	1.20 mg/g DW of betalains	Maximum extraction yield of betalains was obtained at an ethanolic concentration of 50%, citric acid concentration of 1.5%, temperature of 52.52°C, and extraction time of 49.9 min	Lazăr et al. (2021)
Aqueous biphasic systems (ABS) composed of quaternary ammonium‐based ionic liquids and polypropylene glycol	1:10	Extraction of betalains from red beet stems and leaves at a temperature of 20°C for 2 h	6.67 wt% of betalains	The highest amount of betalains was obtained at a temperature of 20°C, a time of 70 min, and a solid‐to‐liquid ratio of 0.12	Rosa et al. (2023)
Water 1% citric acid 0.5% citric acid 0.2% citric acid 0.1% ascorbic acid 50% ethanol, 20% ethanol 0.5% citric acid and 0.1% ascorbic acid 0.2% citric acid and 0.1% ascorbic acid 20% ethanol and 1% citric acid 20% ethanol and 0.5% citric acid	1:5	Washing and scarping to obtain the beetroot pulp Lyophilization after drying the obtained beetroot pulp at temperatures of 40–45°C for 10–12 h Extraction using the temperatures of 20 and 70°C at pH values of 2.5 and 8	20 mg/g of betanin	The highest amount of betanins was obtained by 0.5% citric acid and 0.1% ascorbic acid	Neagu and Barbu (2014)
Water	1:15–1:45	Mixing the dried beetroot pomace with pure water Extraction in a water bath at different pH ranges of 1.50–5.50 and different temperatures of 30–70°C for 2.50–12.50 min Centrifugation at 10,000 rpm for 10 min Collection of supernatant and keeping it under refrigerated conditions	17.07 mg/L of betacyanins and 15.04 mg/L of betaxanthins	The highest amounts of betacyanins and betaxanthins were obtained at the solid‐to‐liquid ratio of 1:15, temperature of 50.04°C, time of 10 min, and pH value of 2.5	Kushwaha et al. (2018)
Water 30% aqueous ethanol 70% aqueous ethanol, and ethanol (Each solvent was acidified with formic acid 1%)	1:15	Extraction of beetroot powder with solvents Sonication for 1 min Centrifugation for 5 min (13,200× *g* at 4°C) Collection of supernatant Repeating the extraction step with a new portion of the solvent Combining the supernatants	7.24 mg/g dw of betacyanins and 4.03 mg/g dw of betaxanthins recovered by 30% ethanolic solution	30% aqueous ethanol extracted the highest amount of betalain pigments	Kusznierewicz et al. (2021)
Methanolic and ethanolic solutions in different proportions of 0%–80% (Acidified with 1% citric acid solution)	1:200	Mixing the sample with solvent in a vortex for 1 min Extraction in a water bath at different temperatures of 5–30°C for 10–30 min Centrifugation at 3500 rpm for 15 min at room temperature Collection of supernatant and its filtration through a millipore membrane	—	The most betalain concentration was obtained at a temperature of 15°C and a time of 10 min for 20% methanolic solution	Sanchez‐Gonzalez et al. (2013)
Water	—	Washing, peeling, and slicing the red beetroot Blanching at 100°C for 10 min Blending with water in a mixer Extraction at a constant speed of 900–1000, and different temperatures of 30–60°C for 1 h Adjusting the pH of the extracted sample to 4.0–5.0 by citric acid (1 M) Ultrafiltration of the extract	96.3% of betanins	The highest amount of betanins was obtained at the temperature of 40°C	Roy et al. (2004)
15% ethanol	Different ratios of 0.2, 0.6, and 1 (w/v)	Blending the beetroot peels in a mixer for 2 min to obtain a pulp Mixing the pulp with solvent Extraction with a thermostat water bath at different temperatures of 20, 35, and 50°C and different times of 1, 3, and 5 h with stirring at 215 rpm Centrifugation of the extracts for 25 min at 6000 rpm Collection of supernatant	1361 mg/L of betacyanins and 952.5 mg/L of betaxanthins	The highest amount of betalain pigments was obtained at the temperature of 20°C, time of 1 h, and solid‐to‐liquid ratio of 0.8 w/v	Zin, Márki, and Bánvölgyi (2020a)
25% ethanol 50% ethanol 75% ethanol	1:10	Grinding the beetroot peels with a pulverizer Mixing the obtained pulp with solvent Single‐stage batch extraction with a laboratory extractor at different temperatures of 20, 35, and 50°C and different times of 10, 35, and 60 min Centrifugation at 6000 rpm for 25 min Collection of supernatant	165.24 mg/L of betacyanins and 97.56 mg/L of betaxanthins	The highest amount of betalains was obtained at the 25% ethanolic solution, temperature of 50°C, and time of 50 min	(Zin et al., 2021
Water	1:20	Homogenization of lyophilized plant material for 1 min with water Centrifugation of homogenate for 10 min (1500× *g*) Collection of supernatant Re‐extraction of insoluble part with water Combination of the extracts Reaching the final volume of the extract to 15.5 mL	2.9 mg/g dw of betanin in Egyptische Platronde cultivar, 5.2 mg/ g dw of betanin in Forono cultivar, 3.6 mg/ g dw of betanin in Little Ball cultivar, 4.1 mg/ g dw of betanin in Rubia cultivar	—	Kujala et al. (2002)

#### 2.1.1 Effect of solvent type

While water can be used to extract red beet betalains (Silva et al., 2020), it can make the separation of betalains challenging. To overcome this problem, the recovery of betalains can be improved by adding ethanol or methanol to water (Bastos & Gonçalves, 2017a; Fu et al., 2020). To extract betalain pigments thoroughly, it is crucial to use methanolic or ethanolic concentrations ranging from 20% to 50% (v/v) (Zin, Márki, et al., 2020a). Notably, low‐polarity hydro‐alcoholic solutions are more effective than polar solvents for betalain extraction because of the weak electrostatic interactions in these pigments (Fernando et al., 2021). The increased extraction efficiency of betalains may also be attributed to the inhibitory effect of hydro‐alcoholic solutions on endogenous enzymes compared to water (Bastos & Gonçalves, 2017b). Furthermore, ethanol can increase the yield of low‐molecular‐weight compounds, such as betalains, by reducing the co‐extraction of pectin and other fibers (Fernando et al., 2021). The addition of ethanol also facilitates the extraction of betalains by reducing the water's polarity (Righi Pessoa da Silva et al., 2018).

However, it should be noted that pure ethanol or high ethanolic concentrations may not be suitable for extracting the bioactive compounds (Righi Pessoa da Silva et al., 2018). The yield of betalain‐rich extract of red beet decreases at ethanol‐to‐water ratios of greater than 1:1 (Iahtisham‐Ul‐Haq et al., 2020). Righi Pessoa da Silva et al. (2018) found that using 100% ethanol to extract red beet betalains reduced the betacyanin and betaxanthin contents (Righi Pessoa da Silva et al., 2018). Roriz et al. (2017) reported that ethanolic concentrations above 20% are unsuitable for extracting betacyanins (Roriz et al., 2017). Moreover, Ravichandran et al. (2013) declared that among distilled water and ethanolic solutions of 30%, 50%, and 70%, ethanol 30% (v/v) recovered the most betalain content (Righi Pessoa da Silva et al., 2018).

Recently, green extractants like eutectic solvents have gained attention for extracting various natural compounds, including betalains (Hernández‐Aguirre et al., 2021). In one study, two natural and acidic deep eutectic solvents (DESs) of magnesium chloride hexahydrate: urea and two natural and acidic aqueous solutions were employed to extract betalains from beetroot waste. The results revealed that the acidified DES extracted the highest amount of betalains, particularly betacyanins, whereas it reduced the betaxanthins content. This phenomenon can be attributed to the betacyanin stability in the extracts and DES selectivity by betacyanin (Hernández‐Aguirre et al., 2021).

#### 2.1.2 Effect of acidification

This study highlights the importance of acidification on red beet betalains concentration. The concentration of betalains decreases with an increase in pH value. Acidification is a viable strategy to enhance the betalain concentration in extracts (Halwani et al., 2018; Hernández‐Aguirre et al., 2021). According to Singh et al. (2017), aqueous extraction of red beet betalains within the pH range of 3–5 recovered more betalain pigments (Singh et al., 2017). Furthermore, Neagu and Barbu (2014) reported that while acidification at low temperatures enhances the extraction yield of beetroot betanins, its extractability is not significantly affected at high temperatures (Neagu & Barbu, 2014).

In one study, ethanolic solutions acidified with citric acid were employed to extract betalains from beetroot. The stability of betalain pigments within the pH range of 3–7 was a critical factor in this study (Lazăr et al., 2021). It was found that acidification was effective in increasing the recovery of betacyanins, primarily by inhibiting their oxidation by polyphenol oxidases (Ravichandran et al., 2013). Moreover, another study revealed that beetroot betanin can be extracted more effectively by acidified solvents than by hydro‐alcoholic solutions. The solution containing 0.5% citric acid and 0.1% ascorbic acid was the most efficient solvent for extracting beetroot betanins (Neagu & Barbu, 2014).

#### 2.1.3 Effect of temperature and time

Heat treatment may degrade betanin pigments through isomerization, decarboxylation, or cleavage (Neagu & Barbu, 2014). Additionally, thermal processing in the presence of oxygen may alter the color of betalain pigments to yellow‐orange through dehydrogenation and decarboxylation (Zin & Bánvölgyi, 2021). Due to the heat sensitivity of betalain pigments (Zin, Anucha, et al., 2020b), it is necessary to use lower extraction temperatures to achieve higher betalain yields from red beet (Ravichandran et al., 2013). Furthermore, while high temperatures reduce the betaxanthin content, they do not have a significant effect on the betacyanin content (Righi Pessoa da Silva et al., 2018). In one study, by elevating the extraction temperature at pH values of 3, 5, and 7, beetroot betalains decreased by 40%. This can be attributed to betaxanthins' higher sensitivity to temperature than betacyanins (Sanchez‐Gonzalez et al., 2013). Another study reported that the highest concentration of red beet betanins was recovered using citric acid solution and water at temperatures of 40 and 50°C, respectively (Halwani et al., 2018). According to Lazăr et al. (2021), extraction of beetroot betalains at 73.54°C led to the degradation of these pigments (Lazăr et al., 2021). Moreover, Zin et al. (2021) reported that when the extraction temperature of red beet betalains was increased to 50°C, the degradation rate of these pigments increased by three‐fold for every 10°C temperature rise (Zin & Bánvölgyi, 2021). Kushwaha et al. (2018) found that the highest betacyanin and betaxanthin contents of red beet were obtained at 60°C (Kushwaha et al., 2018).

Extraction time is another factor that affects extraction efficiency. Long extraction times can cause phytochemicals to decompose and oxidize due to prolonged exposure to adverse environmental factors (Kushwaha et al., 2018). While shorter extraction times may result in partial extraction of betalains, longer extraction times may degrade these pigments (Righi Pessoa da Silva et al., 2018). According to Righi Pessoa da Silva et al. (2018), extending the extraction time only improves the extraction yield of betacyanins (Righi Pessoa da Silva et al., 2018). Zin et al. (2021) found that more betalain content of beetroot was obtained when using a 50% ethanolic solution and increasing the extraction time from 30 to 60 min (Zin et al., 2021). Previous studies also revealed a decrease in the betalain content of red beet during high temperatures and long extraction times (Cardoso‐Ugarte et al., 2014; Halwani et al., 2018; Neagu & Barbu, 2014).

### 2.2 Novel extraction methods

Novel extraction methods used for the extraction of red beet betalains are presented in Table 2.

**TABLE 2:** Novel extraction techniques used for the extraction of red beet betalains.

Extraction method	Solvent	Solid: Liquid ratio	Extraction conditions	Results		References
				Betalain concentration	Optimum conditions	
Enzyme‐assisted extraction	Acetate buffer containing the multi‐component enzyme mix with dosages of 10, 18, 25, 38, and 50 U/g	1:15	Washing, draining, and mixing the red beet samples Using the resulting puree for the extraction of betalains Extraction at temperature of 25 or 45°C and the pH of 5.5 ± 0.1 up to 5 h Filtration of the mixture	11.37 mg/L of betaxanthins and 14.67 mg/L of betacyanins	Enzymatic total dose of 25 U/g, temperature of 25°C, and processing time of 240 min	Lombardelli et al. (2021)
Microwave‐assisted extraction	Ethanol: water (1:1)	0.1:25	Extraction at different powers of 400, 800, and 1200, duty cycles of 50, and 100%, time of 0–160 s Adjusting the volume to 25 mL Cooling in an iced water bath	128.68 mg/100 g of betanins, and 101.41 mg/100 g of betaxanthins	The combination of 400 W and 100% duty cycle for 90–120 s resulted in the highest amount of recovered betanins; whereas at 140–150 s the highest amount of betaxanthins was obtained	Cardoso‐Ugarte et al. (2014)
Microwave‐assisted extraction	Pure water Acidified water 15% (v/v) ethanol‐water Acidified ethanol‐water (0.5% (w/v) ascorbic acid was applied for acidification)	0.1–0.2 (w/v)	Microwave‐assisted extraction at microwave power of 100–800 W for 30–150 s	202.08 mg/100 g of total betalains, 115.89 mg/100 g of betacyanins, and 86.21 mg/100 g of betaxanthins	Extraction with microwave power of 800 W, irradiation time of 150 s by pure water solvent	Zin and Bánvölgyi (2021)
Microwave‐assisted extraction	Citric acid (pH = 5.20) Ethanol (pH = 4.74)	1:5	Microwave irradiation followed by extraction for 10 min Centrifugation of the extracted solution for 5 min at 2500 rpm	229.264 mg/L of betanin in citric acid solution, and 472.113 mg/L of betanin in ethanolic solution	The optimized extraction condition with a citric acid solution was a microwave power of 224.61 MW for 57.06 s, and with an ethanolic solution was a microwave power of 384.25 MW for 74.91 s	Singh et al. (2017)
Microwave‐assisted extraction	Water: ethanol acidification	1:20	After treatment centrifuged at 6000 rpm for 10 min.	The highest betacyanin content is 175–200 mg/L	The optimized extraction condition was 1.5% citric acid, 0.17 duty cycle, 230 W microwave power, 4 min extraction time, and process time 0.5 min	Hazervazifeh et al. (2023)
Pulsed electric field	Deionized water (pH 5.8)	1:5	Incubation without agitation for 1 h at 22°C in the dark Filtration of the extract through a filter paper in a glass funnel	The optimized condition resulted in the extraction of total betalains up to 70%	Using 3 pulses (1 Hz) with a duration of 100 μs, and pulse strength of 2 kV/cm	Visockis et al. (2021)
Pulsed electric field	Phosphate buffer at pH = 6.5	Beetroot samples (8 cylinders)	After PEF treatment, cylinder samples were dried on filter paper	Betanin by 329%, vulgaxanthin by 244%	Different electric field strengths (4.38 and 6.25 kV/cm), pulse number 10–30, and energy input 0–12.5 kJ/kg	Nowacka et al. (2019)
Pulsed electric field	Distilled water	1:20	Extraction was carried out without exposure to light and external oxygen access in the temperature range of 30–80°C.	—	100 μs pulses with electric field strength E = 375–1500 V/cm and total treatment time *t* = 0–0.2 s	Loginova et al. (2011)
Pulsed electric field	Deionized water	—	After treatment for 1 min to obtain a homogenous mash without any addition of water	Increase betacyanin content from 9.29 to 26.61 mg/100 g FW and betaxanthin content from 4.38 to 9.18 mg/100 g FW	1.5 kV/cm electric field strength, 0.66 μF, and 20 pulses, the treatment time was 0.3 ms	Kannan (2011)
Pulsed electric field	Deionized water	Disks of tissue slices, 1 mm in thickness and 4 mm in diameter	Samples after treatment placed in a drop of deionized water. They were then kept in a freezer at 24°C for 4–5 days, and thawed at room temperature for 3 h.	Extraction yield of betalains and ionic species only up to an extraction level of 60%–80%.	Subjected to 270 rectangular pulses of 10 μs at 1 kV/cm field strength, with an energy consumption of 7 kJ/kg, the samples released about 90% of total red coloring and ionic content following 1 h aqueous extraction	Fincan et al. (2004)
Ultrasound‐assisted extraction	Water	1:25	Using different temperatures of 30, 45, and 60°C in the extraction times of 30 and 60 min, with a power of 165 W and a frequency of 25 kHz	4.45 mg/g of betacyanins and 2.42 mg/g of betaxanthins	The highest betalain content was obtained at the temperature of 30°C and the time of 30 min	Silva et al. (2020)
Ultrasound‐assisted extraction	Water Aqueous ethanol solutions (80% v/v) Aqueous β‐cyclodextrin solutions (1 and 5% w/v) Ethanolic solution of β‐cyclodextrin (1% and 5% w/v)	1:10	Stirring the mixture for 3 h Placing the samples in an ultrasonic bath (28 kHz, 80 W, 30 min, without external heating) Centrifugation of the extracts at 7000 rpm for 10 min Filtration Lyophilization of the extracts	2.243 mg of betanin	Extraction at 30 min, 28 kHz, and 80 W	Tutunchi et al. (2019)
Ultrasound‐assisted extraction	Water 20%, 30%, 50% v/v ethanolic or methanolic solutions	1:25	Sonication at 44 kHz for 30 min at 30°C	Betalain values of 0–3.06 mg/g in four cultivars of beetroot	30% ethanol was the most suitable solvent combination for betalain extraction	Fernando et al. (2021)
Ultrasound‐assisted extraction	Deep eutectic solvents (DES) using magnesium chloride hexahydrate and urea in proportions (1:1) and (2:1)	1:30	Mixing the Fresh beet pieces with the DES in a blender. Ultrasonic assisted extraction of betalains at 25 °C for 3 h Vortex agitation for 900 s. Separation of the liquid from the beetroot mass by filtration	3.99 mg/g of total betalains	DES (2:1) was used to extract betalains from beetroot waste	Hernández‐Aguirre et al. (2021)

#### 2.2.1 Pulsed electric field processing

PEF technology generates strong electric fields between two electrodes by rapid and high‐voltage delivery of pulses, which can puncture cell membranes and form pores (Echegaray et al., 2022; Maza et al., 2020). This phenomenon, known as electroporation, facilitates the release of phytochemicals from intracellular to extracellular environments (Carreón‐Hidalgo et al., 2022). Electroporation of cell membranes can be achieved at electric field strengths below 10 kV/cm and low specific energies less than 10 kJ/kg (Ongkowijoyo et al., 2018).

Like other methodologies, this method has both advantages and disadvantages. In terms of advantages, it increases the quality of the product by preserving its nutritional value, sensory attributes, and health‐promoting properties (Fu et al., 2020; Visockis et al., 2021). Moreover, PEF is a safe method that is characterized by short extraction times, low energy consumption, and selective recovery of bioactive compounds (Bocker & Silva, 2022; Visockis et al., 2021). However, this method's drawbacks include the potential corrosion of electrodes and the possible ingress of these substances into the food. This issue can be solved by employing stainless steel electrodes (Fu et al., 2020). This method comes with other drawbacks, such as the high cost of equipment and the possibility of enzymatic degradation of extracted pigments (Bocker & Silva, 2022; Figure 2).

Nowacka et al. (2019) reported that higher betalain content recovered with PEF technology improved the extract's color significantly. The application of PEF processing to beetroot led to a three‐fold increase in betanin extraction. The extraction of betanin and vulgaxanthin from beetroot increased by 329% and 244% when treated with an intensity of 4.38 kV/cm (Nowacka et al., 2019). Loginova et al. (2011) found that the aqueous extraction of betalains from red beets by PEF was accelerated by elevating the temperature to approximately 60°C. However, heat treatment at 80°C for about 1 h caused the complete degradation of betalains (Loginova et al., 2011). According to Kannan (2011), PEF processing resulted in a 38.5% increase in betalain‐rich extract of beets (Kannan, 2011). Çoban et al. (2024) reported that using PEF technology, the maximum content of red beet betalains was obtained at a voltage of 4.6 kV, a pressure of 400 mbar, and a moisture content of 30.90% (Çoban et al., 2024).

#### 2.2.2 Ultrasound‐assisted extraction

UAE is a viable method for extracting bioactive compounds (Ongkowijoyo et al., 2018). Combined with other advanced extraction methods, such as supercritical carbon dioxide extraction and microwave treatment, UAE offers superior efficiency compared to conventional extraction methods like maceration and magnetic stirring. Moreover, this sustainable method has other advantages, including low solvent usage, reduced energy consumption, and short extraction times. Since this method applies moderate temperatures, it is suitable for extracting heat‐sensitive compounds (Fernando et al., 2021). However, the high cost and the possibility of undesirable molecular changes are the main drawbacks of the UAE method (Figure 2; Mehta et al., 2022). Ultrasound‐induced cavitation simplifies the breakdown of plant cell walls, which releases betalains and phenolics into the solvent. Furthermore, the moderate temperature of the UAE method makes it suitable to extract heat‐labile compounds (Fernando et al., 2021).

Fernando et al. (2021) declared that 30% (v/v) ethanol was the most suitable solvent for the UAE of betalains and polyphenols from dried red beetroot powder (Fernando et al., 2021). Silva et al. (2020) reported that the optimal conditions for the UAE of red beet betalains were 30°C and 30 min (Silva et al., 2020). Tutunchi et al. (2019) assessed the impact of oligosaccharides, such as beta‐cyclodextrin, on enhancing the efficiency of UAE of betalains. Their findings showed that the extraction efficiency of betalains was improved by using 5% beta‐cyclodextrin and ultrasound, in comparison to other extraction methods. This enhancement was attributed to the beta‐cyclodextrin's affinity for binding and interacting with betalains (Tutunchi et al., 2019).

#### 2.2.3 Microwave‐assisted extraction

This novel method is based on the principle of cell disruption caused by phenomena, such as dipole rotation and ionic conduction, which increases heat and internal pressure within plant cells (Rodríguez‐Sánchez et al., 2017). Furthermore, the generation of non‐ionized electromagnetic waves within the frequency of 300 to 300,000 MHz, increases the extraction efficiency of bioactive compounds (Echegaray et al., 2022). Reducing solvent requirements, expediting the extraction process, and energy efficiency are among the advantages of the MAE method (Fu et al., 2020). Despite its advantages, the MAE method has disadvantages, including non‐uniform heating and reduced quality of recovered bioactive compounds (Figure 2) (Hazervazifeh et al., 2023). This promising method has been used to obtain extracts rich in flavonoids, anthocyanins, betalains, and carotenoids (Cardoso‐Ugarte et al., 2014).

Cardoso‐Ugarte et al. (2014) reported that the highest extraction efficiency of betalains can be achieved by using the microwave at 80°C, outperforming the conventional solvent extraction at the same temperature (Cardoso‐Ugarte et al., 2014). Singh et al. (2017) declared that the MAE of betalain pigments was improved using citric acid solution and ethanol. The optimal extraction conditions for the citric acid solution and ethanol were microwave powers of 224.61 and 384.25 MW, and durations of 57.06 and 74.91, respectively (Singh et al., 2017). Moreover, Hazervazifeh et al. (2023) reported that the highest betacyanin content was obtained at a microwave power of about 200 W (Hazervazifeh et al., 2023).

#### 2.2.4 Enzyme‐assisted extraction

The EAE method enhances the extraction efficiency of natural pigments by hydrolyzing plant cell walls (Lombardelli et al., 2020). This sustainable method has advantages, such as low extraction temperatures, short extraction times, reduced energy utilization, and little solvent consumption (Fernando et al., 2021). Nevertheless, the high cost of the biocatalysts is the main drawback of the EAE method (Figure 2; Lombardelli et al., 2022). Lombardelli et al. (2021) developed an enzymatic mixture that consisted of pectinase, cellulase, and xylanase based on the composition of red beet cell walls. They reported that the enzymatic hydrolysis of the cell wall occurred more rapidly at 45°C than at 25°C. Furthermore, to achieve the maximum recovery of betalain content, it was most effective to consume an enzymatic range of 10 and 50 units/g (Lombardelli et al., 2021).

### 2.3 Applications

Recently, the use of synthetic food colorants has lost popularity among consumers. Therefore, it has become significant to use foods containing natural pigments, such as betalains in fruits and vegetables (Silva et al., 2020). The Food and Drug Administration (FDA) has approved using betanin (Beetroot Red; EEC No. E 162) as a natural red colorant. It is important to note that betanin has no genotoxic or mutagenic properties. This natural pigment is promising for incorporation into various food products, such as jelly, ice cream, candy, fruit juice, and other similar items (Sadowska‐Bartosz & Bartosz, 2021). Moreover, red beet components can be applied to develop biodegradable packaging films and energy drinks with antioxidant properties (Ravichandran et al., 2013). Manoharan et al. (2012) reported that the strawberry‐flavored ice cream was successfully colored with beet juice as a natural colorant, without affecting its taste or quality (Manoharan et al., 2012). Furthermore, Sruthi et al. (2014) declared that adding red beet betalain‐rich powder at concentrations of 0.2% in ice sherbets and 0.3% in jams resulted in sensory properties similar to those achieved by adding 0.1% carmine, a synthetic red colorant (Sruthi et al., 2014). In one study, adding up to 10% of red beetroot powder effectively prevented food quality deterioration and microbial contamination while improving its sensory attributes, physical properties, and color (Alshehry, 2019). Another study presented that enriching biscuits and cookies with 5 and 10 g of beetroot powder improved the nutritional and sensory characteristics (Amnah, 2013). Furthermore, Ghasempour et al. (2020) reported that incorporating red beetroot extract (0.1/0.2%) and basil seed gum (0.2/0.4%) into probiotic yogurt formulations simultaneously increased physicochemical properties, probiotic viability, antioxidant activity, and product stability (Ghasempour et al., 2020). Moghaddas Kia et al. (2020) incorporated red beetroot extract (0.1% or 0.3%), as an acid‐stabilized natural color, in gummy candies formulation. This study revealed that adding red beetroot extract increased the antioxidant properties of the candies by approximately 50% (Moghaddas Kia et al., 2020).

## 3 PHYSICOCHEMICAL PROPERTIES OF RED BEET BETALAIN‐RICH EXTRACT

The physicochemical properties of red beet juice are influenced by the treatments applied in its manufacturing (Juszczak et al., 2010). Total phenols, total soluble solids (TSS), antioxidant activity, and color are some of the physicochemical properties of the red beet betalain‐rich extract.

### 3.1 Total phenols

The extraction efficiency of low‐molecular‐weight compounds like polyphenols can be improved by ethanol, which reduces the co‐extraction of pectin and proteins. Ethanolic concentrations over 30% are not efficient for extracting polyphenols from red beet. Fernando et al. (2021) reported that 30% ethanol recovered 19.3% and 71% more polyphenols from red beet than water and pure ethanol, respectively (Fernando et al., 2021).

Lazăr et al. (2021) reported that while the extraction of phenolic compounds from red beet was negatively affected by the temperature and time, it was positively affected by the concentrations of ethanol and citric acid. Additionally, increasing the extraction time and citric acid concentration simultaneously reduced the yield of polyphenols (Lazăr et al., 2021). In one study, pre‐treatment with enzymes, such as cellulase and pectinase before the extraction process recovered more polyphenols from red beet than maceration (Fernando et al., 2021). Moreover, Righi Pessoa da Silva et al. (2018) declared that the UAE method recovered higher amounts of phenolics from red beet than the conventional solvent extraction (Righi Pessoa da Silva et al., 2018).

### 3.2 Total soluble solids

TSS is measured to determine the sugar content and the amount of monosaccharides and disaccharides in juices. TSS also indicates the quantity of dissolved ingredients in the extract. Different cultivars of beetroot exhibit varying TSS levels. The growing system and water stress may also influence the TSS levels of beetroot (Šlosár et al., 2020). In one study, membrane clarification of red beet juice reduced its TSS content. The initial and clarified TSS contents of red beet juice were 12° and 6.4°, respectively. Furthermore, while betacyanin and betaxanthin contents in the initial juice were 44.35 and 30.98 mg/L, after clarification, these quantities were 41.73 and 26.94 mg/L, respectively (Amirasgari & Mirsaeedghazi, 2015). Akan et al. (2022) found that the storage of fresh‐cut red beets increased the TSS content from 10.40% to 13.40%. Moreover, PVC‐packed red beets lost more TSS than PET‐packed ones during storage. This reduction in PVC‐packed samples may be attributed to the breakdown of sugars during respiration (Akan et al., 2022).

### 3.3 Antioxidant activity

Conventional solvent extraction using water and organic solvents can be applied to recover antioxidant compounds from plant tissues. However, novel extraction methods are more effective than conventional solvents in enhancing antioxidant activity (Righi Pessoa da Silva et al., 2018). Ravichandran et al. (2013) evaluated the DPPH radical scavenging activity of red beet betalains under various treatment conditions. They reported that boiling, roasting, and microwave treatments increased the antioxidant activity by 3‐fold, 3‐fold, and 2‐fold, respectively (Ravichandran et al., 2013). In one study, UAE of red beet betalains and polyphenols yielded higher antioxidant activity than conventional solvent extraction (Righi Pessoa da Silva et al., 2018).

According to Zin et al. (2021), the antioxidant activity of red beet polyphenols is significantly affected by temperature (Zin et al., 2021). Pandey et al. (2018) assessed the impact of different temperatures (10, 20, 30, 40, and 50°C) on the antioxidant activity of red beet. The results demonstrated that the highest and the lowest antioxidant activities were obtained at 40 and 10°C, respectively. Moreover, the antioxidant activity of red beet was associated with the betalain content, phenolics, and antioxidant compounds released during the thermal treatment (Pandey et al., 2018).

The solid‐to‐liquid ratio also affects the antioxidant activity. According to Guine et al. (2018), extraction of red beet betalains using 50% (v/v) ethanol and 60% (v/v) acetone at a solid‐to‐liquid ratio of 0.2, reduced the antioxidant activity by 7% and 12%, respectively (Guine et al., 2018).

### 3.4 Color

HunterLab colorimeter measures the color of the extract (Amirasgari & Mirsaeedghazi, 2015). The L* value shows the sample's brightness or darkness. Moreover, the a* and b* values indicate the color direction ranging from red to green and yellow to blue, respectively (Fernando et al., 2021). While the a* value correlates with betalain content positively, the L* and b* values correlate negatively (Prieto‐Santiago et al., 2020). The positive correlation between L*a*b values and betalain content has also been confirmed in previous studies (Fernando et al., 2021). The main factor for assessing color degradation is the a/b ratio (Chandran et al., 2014).

**FIGURE 3:**
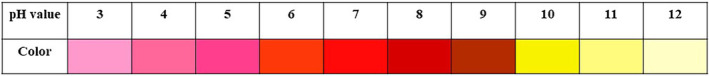
Color changes of red beet betalain‐rich extract at different pH values.

There is an association between color and thermal degradation of red beet betalains (Prieto‐Santiago et al., 2020). Betalains may undergo dehydrogenation and decarboxylation during thermal processing, resulting in yellow‐orange color variation (Zin & Bánvölgyi, 2021). Additionally, pH changes may affect the color (Phuhongsung et al., 2020). The hue of red beet betalains changes from pink to red within the pH range of 3 to 7 (Antigo et al., 2017). At pH values greater than 7, betanin pigments are hydrolyzed into betalamic acid and cyclo‐dopa‐5‐O‐glucoside, yielding a yellowish‐brown hue (Zin & Bánvölgyi, 2021; Figure 3). According to Attia et al. (2013), 7% color loss in red beet juice is obtained within the pH range of 3–7. However, color losses at pH values of 8 and 10 were 21.87% and 50%, respectively (Attia et al., 2013). Temperature also affects the color significantly (Prieto‐Santiago et al., 2020). In one study, elevating the temperature reduced the reddish color of red beet juice. Moreover, increasing the temperature from 25 to 45°C resulted in 45.5%–72.7% color loss due to the degradation of red beet betacyanins (Kayın et al., 2019).

## 4 BIOLOGICAL ACTIVITIES OF RED BEET BETALAINS

Red beet is one of the important vegetables containing vitamins, minerals, flavonoids, triterpenes, steroids, saponins, sesquiterpenoids, coumarins, betalains, carotenoids, and alkaloids (Beals et al., 2017). The health‐promoting attributes of red beet derive from these compounds individually or through their synergistic interactions (Hadipour et al., 2020). Some of the biological activities of red beet betalains have been reviewed here.

### 4.1 Antioxidant activity

The antioxidant activity of red beet betalains is influenced by their structure. The high antioxidant properties of betalains are attributed to their capacity to donate electrons. Additionally, the antioxidant activity is enhanced by the presence of phenolic hydroxy groups and cyclic amines in their molecular structure (Sadowska‐Bartosz & Bartosz, 2021).

Extraction parameters, including solid‐to‐liquid ratio, temperature, time, and pH play significant roles in the antioxidant activity of red beet betalains. According to Kushwaha et al. (2018), increasing the extraction temperature from 40 to 55°C enhanced the antioxidant activity. However, the antioxidant activity was reduced at the temperature range of 55–60°C due to the degradation of phenolic and betalain compounds. While extending the extraction time increased the antioxidant activity significantly, the effect of pH was not significant (Kushwaha et al., 2018). In one study, the maximum antioxidant activity of red beet betalains and polyphenols was obtained at 40°C (Pandey et al., 2018). Lechner et al. (2010) reported that treatment of rats with N‐nitrosomethylbenzylamine (NMBA) and regular water containing 78 mg of commercial red beet dye led to a 45% reduction in the number of esophageal papillomas. Moreover, cell proliferation was decreased and no toxicity was observed after 35 weeks of consumption. These beneficial effects could be attributed to the antioxidant properties of beetroot betanin (Lechner et al., 2010). Sakihama et al. (2023) found that red beet betalains demonstrated antioxidant activity against peroxynitrite in cultured mouse fibroblasts (Sakihama et al., 2023).

### 4.2 Anti‐inflammatory properties

Inflammation, a response to body injury or infection, is associated with cellular destruction and tissue damage. Betalains inhibit the activity of inflammatory‐inducing enzymes, such as lipoxygenase (LOX) and cyclooxygenase (COX). Increasing the concentration of betanidin enhances the anti‐inflammatory response and prevents tumor formation (Carrillo‐López & Yahia, 2017). Moreno‐Ley et al. (2021) reported that betalains can reduce several inflammatory markers, including Tumoral Necrosis Factor α (TNF‐α), Interleukins (IL‐1β), and Nuclear Factor (NF‐κB) (Moreno‐Ley et al., 2021). Imamura et al. (2022) found that red beetroot betalains can mitigate the development of Alzheimer's disease by inhibiting the aggregation of amyloid‐β (Aβ), which is a primary causative factor in this disease (Imamura et al., 2022). Adhikari et al. (2017) reported that oral consumption of beetroot extract at doses of 100 and 200 mg/kg inhibited the carrageenan‐induced rat paw edema by 26.9% and 34.6%, respectively (Adhikari et al., 2017).

### 4.3 Anti‐cancer properties

Oxidative stress, a key factor in the pathophysiological progression of cancer, can be potentially reduced by bioactive compounds of red beet, such as phenolics, flavonoids, betaxanthins, and betacyanins. The remarkable antioxidant properties of red beetroot are associated with its anti‐cancer efficacy by altering the metabolism of cancer cells (El‐Beltagi et al., 2018). Betanin is a growth inhibitor for breast, colon, stomach, and lung cancer cells. Its effectiveness is within the concentration range of 12.5–200 μg/mL (Carrillo‐López & Yahia, 2017). Kapadia et al. (2011) reported that beetroot extract exhibited anti‐cancer activity at low concentrations of 25 and 78 μg/mL in mice and rats, respectively (Kapadia et al., 2011). Moreover, El‐Beltagi et al. (2018) found that the survival rate of lung cancer cell lines (A549) decreased by increasing the concentration of red beetroot ethanolic extract (El‐Beltagi et al., 2018). Saber et al. (2023) declared that beetroot hydro‐alcoholic extract reduced the proliferation and growth of colon cancer cells (HT‐29 and Caco‐2) by modulating the essential genes without any detrimental effects on normal cells (Saber et al., 2023).

### 4.4 Antimicrobial activity

The antimicrobial potential of betalains can be attributed to their impact on the structure, function, and permeability of microorganism cell membranes. Phenolic compounds may cause cell death in bacteria through cellular pH gradient disruption, reduction in ATP levels, and loss of proton motive force (Čanadanović‐Brunet et al., 2011).

El‐Beltagi et al. (2018) found that beetroot extract showed an antibacterial effect against a broad spectrum of foodborne pathogens. Gram‐positive bacteria, including *Bacillus, Micrococcus, Staphylococcus* and *Streptococcus* were more susceptible to beetroot extract than gram‐negative bacteria like *Escherichia coli* and *Pseudomonas aeruginosa*. This can be explained by the high content of phenolic compounds in red beetroot extract, disrupting the cell wall structure of gram‐positive bacteria (El‐Beltagi et al., 2018). Furthermore, a thick peptidoglycan layer covalently linked to the teichuronic and teichoic acids of the cell walls of gram‐positive bacteria renders them less resistant to these antimicrobial compounds (Salamatullah et al., 2021).

### 4.5 Anti‐diabetic activity

Diabetes elevates blood glucose levels by reducing the rate of total glucose metabolism in the body, a condition known as hyperglycemia (Hadipour et al., 2020). Bioactive compounds can mitigate postprandial hyperglycemia by modulating glucose digestion, absorption, and transport (Aliahmadi et al., 2021). Aliahmadi et al. (2021) found that oral consumption of 100 g of raw beetroot in conjunction with medications, such as metformin and/or glibenclamide, had beneficial effects on 44 diabetic patients (Aliahmadi et al., 2021). Abd El‐Ghffar et al. (2019) found that treating diabetic rats with *Beta vulgaris* L. extract reduced the serum glucose levels, lipid profile, and atherogenic risk. This intervention had no mortality or adverse effects (Abd El‐Ghffar et al., 2019).

### 4.6 Hepatoprotective activity

The liver, the body's main organ of metabolic processes, plays pivotal roles in plasma protein synthesis, detoxification, and red blood cell breakdown. Phenolic compounds augment the activity of antioxidant enzymes by influencing the levels of harmful oxygen radicals in living cells (Sinaga et al., 2020).

Sinaga et al. (2020) assessed the impact of high‐intensity exercise on liver damage and observed an increase in blood flow to skeletal muscles concurrently with a decrease in blood flow to the liver, which can potentially induce damage (Sinaga et al., 2020). Kassem et al. (2020) found that gallic acid, as the main phenolic acid in leaf extracts, exhibits a protective effect against carbon tetrachloride‐induced liver damage and counteracts the ethanol‐induced toxicity (Kassem et al., 2020).

## 5 PURIFICATION OF RED BEET BETALAINS

Simultaneous extraction of compounds with similar polarity is one of the recovery challenges of betalains (Fernando et al., 2022). There are several purification methods for betalains, including flash chromatography (Fernando et al., 2022), ion‐pair high‐speed counter‐current chromatography (IP‐HSCCC; Jerz et al., 2013), high‐performance liquid chromatography (HPLC; Mosquera et al., 2020), adsorbents (Nestora, 2017), and aqueous two‐phase extraction (Rodríguez‐Herrera et al., 2023).

Sawicki, Bączek, et al. (2016) employed solid‐phase extraction and modified QuEChERS methods, followed by micro‐HPLC coupled with a mass spectrometer for recovery and quantification of red beet betalains (Sawicki, Surma, et al., 2016). Nestora et al. (2016) used dipicolinic acid molecularly imprinted polymer as a sorbent for selective solid‐phase extraction of betanin and isobetanin from red beet extracts. Since dipicolinic acid is similar to chromophore group of betanin, it was chosen as a molecularly imprinted polymer to achieve high yields of betanin and isobetanin in single step (Nestora et al., 2016). Rosa et al. (2023) employed aqueous biphasic systems (ABS) composed of quaternary ammonium‐based ionic liquids (ILs) and polypropylene glycol (PPG) for the one‐step extraction and separation of betalain pigments from red beets. In this process, a monophasic aqueous solution of the IL and PPG was used for the extraction, and betalains were subsequently separated into opposite phases. Using affinity resins to separate betalains from the respective phases recovered 96% of betalains (Rosa et al., 2023). In one study, copolymeric adsorbents composed of polyvinylpyrrolidone‐co‐poly(divinylbenzene) (PVP‐co‐PDVB) were employed for the recovery of red beet betalains through solid‐phase extraction technique (Kaba et al., 2020). In another study, an absorbent column packed with Sepabeads SP207 resin was used to recover betalains from *Beta vulgaris* L. hairy roots (Georgiev et al., 2012). Spórna et al. (2010) applied HPLC and IP‐HSCCC to isolate red beetroot betacyanins and their derivatives. In HPLC, betanin and isobetanin were isolated more rapidly than their derivatives, whereas, in IP‐HSCCC, dehydrogenated derivatives were eluted first (Spórna et al., 2010). In one study, high‐performance counter‐current chromatography (HPCCC) was used to isolate betanin and its decarboxylated and dehydrogenated derivatives. In this technique, 2‐decarboxy‐betanin was separated from 17‐ and 2,17‐bidecarboxy‐betanin as well as from neobetanin and betanin (Spórna‐Kucab et al., 2012).

## 6 SAFETY ISSUES

Organic solvents used in conventional extraction of betalains are readily available and cost‐effective. However, since they may have residues in food products, they are associated with food safety concerns (Fu et al., 2020; Rosa et al., 2023). According to Yasokawa et al. (2010), the toxicity of methanol differs from that of ethanol. The probable cause of methanol‐induced toxicity is the conversion of methanol to formaldehyde by exposed cells (Yasokawa et al., 2010). The non‐toxicity of the ethanol makes it a superior solvent to methanol in the food industry (Fernando et al., 2021).

According to Rosa et al. (2023), ILs used for the extraction of bioactive compounds are toxic, which is associated with the specific cations and anions present in the solvent composition (Rosa et al., 2023). Due to the drawbacks of ILs, including high cost, toxicity, and biodegradability issues, efforts have been made to replace them with other environmentally friendly solvents (Alvarez de Cienfuegos et al., 2011; Cao & Su, 2021).

One potential solution to address solvent contamination in food substances is adopting innovative methods like PEF and UAE (Fu et al., 2020). Additionally, EAE, a novel approach that eliminates the need for organic solvents, offers a green strategy for betalain extraction from red beets (Lombardelli et al., 2021). Furthermore, using DESs and NADESs mitigates the disadvantages of ILs. These solvents have lower costs, high biodegradability, and low toxicity (Mehariya et al., 2021; Omar & Sadeghi, 2022).

## 7 CONCLUSION

While water serves as the primary extraction medium for red beet betalains, hydro‐alcoholic solutions ranging from 20% to 50% recover more betalain pigments than water alone. Furthermore, novel extraction methods recover more betalain pigments than conventional solvents. PEF is the most commonly applied for extracting red beet betalains among these methods. It is important to note that the extraction conditions may influence the physicochemical properties of red beets, including total phenolics, TSS, antioxidant activity, and color.

Red beet betalains offer various health‐promoting advantages, including antioxidant, anti‐inflammatory, anti‐cancer, anti‐diabetic, antimicrobial, and hepatoprotective properties. These pigments are gaining recognition for their broad applications in biodegradable packaging films, energy drinks, and natural colorants in the food industry. Consequently, selective recovery of betalain pigments through various chromatography techniques and adsorbents is required for their utilization. Additionally, it is essential to address the safety concerns regarding the extraction solvents for betalains. Adopting novel extraction methods and alternative green solvents represent promising approaches to mitigate these issues.

## CONFLICT OF INTEREST STATEMENT

The authors declare that they have no conflict of interest.

## ETHICAL APPROVAL

This study does not involve any human or animal testing.

## Data Availability

No datasets were generated or analyzed in this study.
